# COVID-19 Neutralizing Antibodies in Breast Milk of Mothers Vaccinated with Three Different Vaccines in Mexico

**DOI:** 10.3390/vaccines10040629

**Published:** 2022-04-18

**Authors:** Olivia Cabanillas-Bernal, Karla Cervantes-Luevano, Gonzalo Isai Flores-Acosta, Johanna Bernáldez-Sarabia, Alexei F. Licea-Navarro

**Affiliations:** Biosafety Laboratory Level 3, Biomedical Innovation Department, Ensenada Center for Scientific Research and Higher Education (CICESE), Ensenada 22860, Mexico; cabanillas@cicese.mx (O.C.-B.); kcervantes@cicese.mx (K.C.-L.); gflores@cicese.edu.mx (G.I.F.-A.); jbernald@cicese.mx (J.B.-S.)

**Keywords:** breast milk, neutralizing antibodies, vaccines, COVID-19, SARS-CoV-2

## Abstract

Severe acute respiratory syndrome coronavirus-2 (SARS-CoV-2) has caused the largest pandemic of this century, and all aspects of this virus are being studied. The efforts to mitigate the negative effects associated with the SARS-CoV-2 pandemic have culminated in the development of several vaccines that are effective and safe for use to the general population. However, one aspect that remains relatively underexplored is the efficacy of different vaccines technologies (mRNA and Adenovirus) in providing passive immunity to infants through breastmilk of vaccinated mothers, and whether the antibodies passed through breast milk are functional. In this study, using a Micro-neutralization assay, we evaluate the presence of neutralizing antibodies in breast milk of lactating mothers vaccinated against SARS-CoV-2 with the Pfizer-BioNtech, Johnson & Johnson (J&J)/Janssen, and CanSino Biologics vaccines. Our results show the greatest neutralizing effect in breast milk from mothers vaccinated with Pfizer, followed by mothers vaccinated with J&J. CanSino vaccinations yielded the breast milk with the least neutralizing effects. The results found in this study relating to the neutralizing capacity of breast milk against SARS-CoV-2 highlight the importance of corresponding health authorities recommending vaccination to lactating mothers and of the continuance of breastfeeding to infants due to the potential health benefits.

## 1. Introduction

In December 2019, the authorities of the city of Wuhan, China, confirmed to the World Health Organization (WHO) the existence of hundreds of cases of a new respiratory disease, and days later they confirmed the existence of a new pathogenic agent of the *Coronaviridae* family. On 28 January 2020, the complete genome of this coronavirus was published, and on 11 February, the WHO named it COVID-19 [1]. However, the International Committee on Virus Taxonomy (ICTV) later formally renamed it as severe acute respiratory syndrome-coronavirus-associated (SARS-CoV-2) [2]. The SARS-CoV-2 has caused the largest pandemic of this century with ~488 million cases and 6.1 million deaths globally (https://www.worldometers.info/coronavirus/, accessed on 31 March 2022). The severity of this pandemic is reflected in the great efforts that have been put in place to mitigate its spread, culminating in the development of several vaccines against the virus in less than a year [3,4,5,6].

Diverse vaccines for COVID-19 have been licensed for emergency use in the United States and other countries, based on different delivery technologies. These include the Pfizer-BioNtech (BNT162b2) [3] and Moderna (mRNA-1273) [4] nucleoside-modified messenger RNA-based vaccines, and the Johnson & Johnson (J&J)/Janssen (Ad26.COV2.S) [5] and CanSino Biologics (Ad5-nCoV) vaccines [6], which are based on modified adenovirus vectors. To date, CanSino vaccine is only available outside the US.

According to the norms established for clinical trials, vaccines against COVID-19 did not include trials in pregnant and/or lactating women, however, national organizations in the United States have recommended and supported vaccination for this group based on experience with other vaccines, since there is no previous evidence that non-live vaccines cause adverse effects in infants through breastfeeding [7]. Furthermore, it has been shown that vaccination can promote passive immunization to the infant and provides a first line of defense against different pathogens; all of this can be achieved through breastfeeding [8]. Although prior data suggests a possible passive immunity against COVID-19 transferred from vaccinated mother to child through breast milk, there is little information on this topic.

The human neonate has an immune system that is not fully developed or functional, and is hypogammaglobulinemic, since just a little antigen exposure occurs in the uterus [8,9]. Due to these limitations, the transfer through breast milk represents the main source of antibodies and a first line of defense against infections for neonates and infants. Secretory IgA (SIgA) represents the main immunoglobulin in breast milk, followed by secretory IgM and IgG, and serves as the first line of defense against mucosal infections [10]. These immunoglobulins effectively prevent microorganisms from entering tissues and are non-inflammatory. The SIgA antibodies in milk occur in large quantities (1 mg/mL) and are directed against all microbes that the mother has previously encountered, providing protection against a wide range of microorganisms, especially those that are likely to be found in the baby’s environment. The antiviral defense functions of SIgA in the mucosa have been well reported for the measles virus [11]. There is also evidence of IgA neutralizing antibodies (NAbs) against respiratory infections such as influenza A virus in human milk [12], suggesting that SIgA from breast milk may protect against the entry of the SARS-CoV-2 virus onto the mucosal surface of the respiratory tract [13].

Until now, most of the studies on the presence of antibodies in breast milk have focused only on the mRNA based vaccines from Pfizer and Moderna [14,15,16,17,18,19,20]. Regarding the studies carried out on breast milk from mothers vaccinated with vaccines other than mRNA, a study conducted with breast milk from mothers who received two doses of an inactivated virus vaccine (CoronaVac, Sinovac) was reported [21]. Another study that has not yet been peer reviewed included a group of mothers vaccinated with J&J [22]. While these previous studies have shown that anti-SARS-CoV-2 IgA and IgG antibodies are present in breast milk of vaccinated mothers, one of the biggest questions that remains is how much protection babies receive from breast milk, and whether the antibodies passed through breast milk are functional to neutralize SARS-CoV-2. In this study, the neutralizing effect of the breast milk of 36 lactating mothers vaccinated against COVID-19 with the Pfizer, J&J, and CanSino vaccines was evaluated. The milk samples were put in direct contact with the SARS-CoV-2 to evaluate if the immunoglobulins passed in the milk could inhibit viral replication. These studies will provide insights on the passive protection conferred to babies by vaccinated mothers, and will help determine which vaccine technology is more effective in eliciting a higher antibody production that is transferable to the infant. Furthermore, this study provides convincing evidence for the WHO and other health authorities to recommend the vaccination of pregnant or lactating mothers due to the potential health benefits.

## 2. Materials and Methods

### 2.1. Study Design

This was a prospective cohort study conducted between June and August 2021 at The Ensenada Center for Scientific Research and Higher Education, Baja California (CICESE). The eligibility criteria included lactating mothers vaccinated with any of the anti-COVID-19 vaccines approved for use in Mexico, and control mothers who had not been vaccinated. Considering this criteria, four groups of nursing mothers were formed; mothers vaccinated with Pfizer, J&J, and CanSino vaccines, and a fourth group including samples of breast milk from unvaccinated mothers. Participants were recruited through social medial, personal communications, lactation consultants, and the assistance of a nongovernmental support center for the health and perinatal education for mothers. Informed consent was obtained from study participants. The age of the mothers ranged from 25 to 36 years old. The infants of the women included in the study had an age range of 0 to 36 months.

### 2.2. Samples Collection and Preparation

Breast milk samples were collected by the donors into sterile containers either by manual expression or using breast pump devices. The samples were collected three to six weeks after the mother’s vaccination schedule had been completed. All samples were personally provided by the mother. Participating mothers donated ~1–2 ounces of fresh or frozen milk. All samples were recruited and stored at CICESE at −80 °C until further processing. 

The samples were thawed at 8 °C, then were centrifugated at 8 °C; 12,000 g for 10 min [23]. The aqueous phase between the cellular pellet and the upper fat phase was carefully separated. All samples were immediately frozen at −80 °C until the neutralization tests. The study was approved by the ethical committee of General Hospital (approval HG-X034-21).

### 2.3. Cell Culture and Viral Strain

All work with live virus was performed in a Biosafety Laboratory Level 3. VERO E6 cells were maintained in DMEM media supplemented with 2 mM L-Glutamine, 10% FBS, and 100 units/mL penicillin-streptomycin at 37 °C in a 5% CO_2_ humidified incubator. For the measurement of SARS-CoV-2 neutralizing antibodies, the B.1 variant MX-BC2/2020 (D614G) isolated in our laboratory (GISAID ID: EPI_ISL_747242) was used. The virus strain was amplified and titrated as previously described (Cervantes et al., under review).

### 2.4. SARS-CoV-2 Neutralization

The presence of neutralizing antibodies to SARS-CoV-2 in breast milk was evaluated by a Micro-neutralization assay, where the cytopathic effect (CPE) prevention was measure with a colorimetric readout as previously described [24], with some modifications. Briefly, breast milk samples were tested in duplicates in 2-fold serial dilutions from 1:10 to 1:1280. In a 96-well U-bottom plate, 55 µL breast milk dilution was mixed in a 1:1 volume ration with a viral dilution containing 0.02 TCID50 SARS-CoV-2 per cell, and the mixture was incubated for 1 h at 37 °C, in a 5% CO_2_ humidified incubator. Then, 100µL of the breast milk-virus mixture was added to a 96-well cell plate containing semi-confluent (40%) VERO E6 monolayer. The plates were incubated for 96 h at 37 °C in a 5% CO_2_ humidified incubator. After 96 h of incubation, CPE prevention by the neutralizing antibodies was evaluated using a colorimetric read out based on neutral red solution. As a positive neutralization control, the serially diluted plasma of a Pfizer vaccinated mother was used.

The 50% neutralization titer (NT50) was determined through two methods: (1) using 4-parameter nonlinear regression (GraphPad Prism 8.0), and (2) selecting the highest dilution that showed an optical density (OD) value greater than the cut-off value (average of the OD values of the uninfected cell control divided by two).

### 2.5. Statistical Analysis

Statistical analyses were conducted in GraphPad Prism Version 8.0, Microsoft Excel 2019, and Statistics Kingdom (https://www.statskingdom.com/, accessed on 31 March 2022) version 2021. Prior to evaluation of comparison of the data, the normality for Log10NT50 was determined by the Shapiro-Wilk normality test (*p* < 0.05). Comparisons of the distribution of the Log10NT50 among breast milk with different vaccination profiles were compared using the Kruskal-Wallis test with multiple comparison and Bonferroni correction (α). The significance level we considered was *p* < 0.05. The effect of size was calculated prior the Kruskal-Wallis test.

## 3. Results

### 3.1. Study Population

A total of 38 breast milk samples were included in the study. The samples were distributed into four groups corresponding to the vaccination profile: Pfizer (N = 11), J&J (N = 17), CanSino (N = 7), and no vaccine (N = 3). All but one (Asian) participants were of white/Hispanic ethnicity. The average age of the mothers was 31 (ranged from 25 to 36 years old). No serious adverse reactions after COVID-19 vaccinations were reported by the donors. One participant (from the CanSino group) reported having been vaccinated while pregnant, while the rest had already delivered at the time of vaccination. No participants were diagnosed with COVID-19 during the sample collections, while eight participants reported a positive COVID-19 test before receiving the vaccines. None of the donors with a previous positive diagnosis of COVID-19 reported a history of hospitalization for this disease. None of the participants reported being under treatment with antibiotics, presenting fever, or a flu-like illness during the collection of the sample.

### 3.2. Neutralizing Antibodies Titer

From the cut-off value, calculated as the average of all the uninfected cell control ODs divided by two, the neutralizing antibodies titer of each analyzed sample was calculated (Table 1). Samples with neutralization titers of at least twice that of the control group with no vaccine (>1:20), were taken as positive for the presence of neutralizing antibodies to SARS-CoV-2. In 73% of the Pfizer samples and 65% of the J&J samples, we observed the capability to neutralize SARS-CoV-2 infectivity in vitro, whereas only 14% of milk samples from the CanSino vaccination profile showed neutralizing potential. Milk from Pfizer and J&J vaccinated mother showed similar titers of neutralizing anti-SARS-CoV-2 antibodies. Breast milk from mothers vaccinated with CanSino showed a neutralizing capacity similar to the no vaccine group. The positive neutralization control showed a neutralizing antibodies titer of 1:320. Regarding the recovered COVID-19 participants, 87.5% (7/8 participants) showed neutralizing antibodies against SARS-CoV-2. 

### 3.3. Statistics

**Figure 1:**
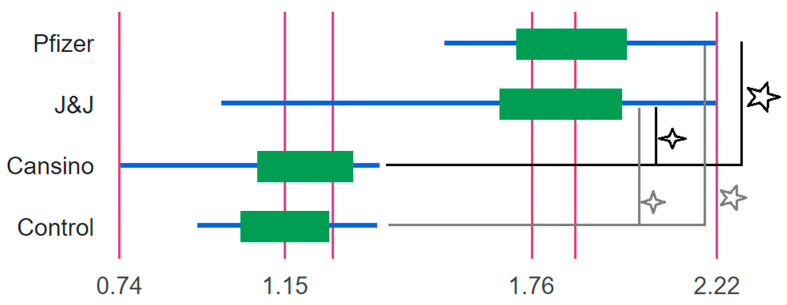
Box plot of the Log10NT50 of the breast milk from mother with different vaccination profiles. All groups were compared with the Kruskal-Wallis test with multiple comparisons. Significant differences (star) were observed in the Log10NT50 between Pfizer, CanSino (*p*-value 0.0006), and the control (*p*-value 0.01). In addition, significant differences (asterisk) were also observed between J&J, CanSino (*p*-value 0.002), and the control group (*p*-value 0.01). For each vaccination Log10NT50 profile the maximum, median, and minimum values are shown.

**Figure 2:**
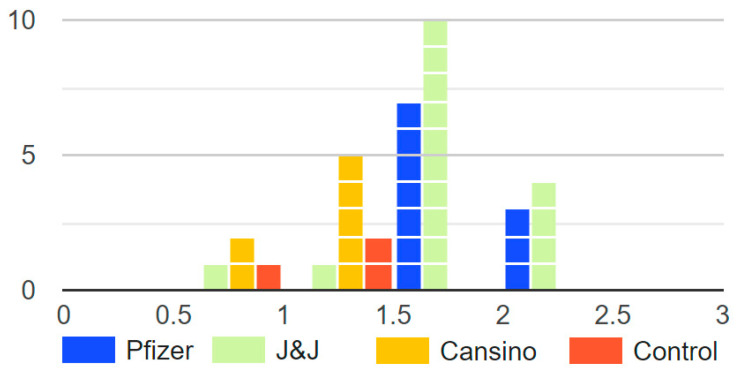
Histogram of the Log10NT50 of the breast milk from mother with different vaccination profiles. The figure displays how only Pfizer and J&J groups had Log10NT50 over 2, and that the control (no vaccine) and CanSino groups had lower Log10NT50.

The NT50 and Log10NT50 titers were calculated using 4-parameter nonlinear regression (GraphPad Prism 8.0). From the groups of breast milk samples analyzed, we observed a clear statistical difference between the Log10NT50 of vaccinated mothers’ samples under the Pfizer and J&J vaccination profile in comparison with CanSino and the non-vaccinated control group (Figure 1). It is important to underscore that there were no statistical differences between the Log10NT50 of vaccinated mothers’ breast milk with the CanSino profile and the control (Figure 1.). The distribution of the Log10NT50 can be observed in Figure 2. The Log10NT50 distribution of Pfizer and J&J was associated with higher values, while the CanSino and no vaccine were associated with lower values. The J&J vaccination profile showed a wider range for the Log10NT50 values. 3.1.

## 4. Discussion

Presently, it is well known that breast milk protects against infections in infants primarily through secretory IgA antibodies [10]. This immunologic response of secretion of antibodies to the breast milk is mainly stimulated when the mother is faced with an infection, or when she receives immunization through the administration of vaccines against some particular pathogens. In the last two years, infection caused by SARS-CoV-2 has caused the largest pandemic of this century. The severity of this pandemic is reflected in the great global efforts to mitigate its spread, which have led to the development of several vaccines against the virus in less than a year [3,4,5,6]. Although vaccines against COVID-19 did not include clinical trials in pregnant or lactating women, this group has been vaccinated throughout the world. This has motivated different research groups to contribute to the knowledge of how passive immunity against COVID-19 is transferred from vaccinated mother to child through breast milk.

In this study, we evaluate the presence of neutralizing antibodies in breast milk of lactating mothers vaccinated against SARS-CoV-2 with the Pfizer, J&J and CanSino vaccines using a micro-neutralization assay. We found that milk produced by mothers vaccinated with Pfizer and J&J vaccines is a source of functional neutralizing antibodies against SARS-CoV-2. The neutralizing antibodies levels were lower in samples from mothers vaccinated with CanSino. A high percentage—73% and 65% of the milk samples corresponding to Pfizer and J&J vaccines, respectively–were able to neutralize SARS-CoV-2 infectivity in vitro, whereas only 14% of milk samples from CanSino vaccinated mothers were able to do so.

The presence of anti-SARS-CoV-2 IgA and IgG antibodies in the breast milk of vaccinated mothers has been conclusively shown for mRNA vaccines [14,15,16,17,18,19,20], and although limited, studies conducted on breast milk from mothers vaccinated with adenovirus-based vaccines also exist and reported the presence of specific IgA and IgG antibodies against SARS-CoV-2 [21,22]. To date investigations on the functionality of the anti-SARS-CoV-2 IgA and IgG remains limited. In addition, there are no studies that investigate and compare the functionality of IgA and IgG anti-SARS-CoV-2 from lactating mother vaccinated with mRNA vaccines and modified adenovirus-based vaccines. In a study conducted with 18 lactating women with a positive test result for COVID-19 (not vaccinated), 62% of the collected milk samples were found to neutralize SARS-CoV-2 infection in vitro [25]. These studies suggest that the milk produced by mothers infected by the virus is a source of anti-SARS-CoV-2 antibodies with virus-neutralizing capacity, and highlights the importance of studying whether the antibodies present in the breast milk of mothers vaccinated against COVID-19 are also functional. Since the presence of anti-SARS-CoV-2 IgA and IgG antibodies in the breast milk of vaccinated mothers has been conclusively shown, the aim of this work was to contribute to the knowledge in this area, studying whether the antibodies passed from mother to child through maternal milk are functional. Our data complements the results previously reported [25] by showing that in addition to mothers infected with COVID-19, mothers vaccinated against the virus are capable of producing anti-SARS-CoV-2 neutralizing antibodies.

Although our study was limited by a small number of samples and different sample sizes between groups, our data suggest a potential immune benefit against COVID-19 for breastfed infants, and that the Pfizer and J&J vaccines elicit better passive immunity with neutralizing antibodies for SARS-CoV-2 in breast milk, compared to the CanSino vaccine. The difference observed in neutralization effect of each vaccine against the virus is in accordance with the reported efficacy of each vaccine. The Pfizer vaccine showed the highest efficacy in trials (95%), followed J&J (71–81%), and then CanSino (65%) [3,5,6]. Therefore, we speculate the neutralizing capacity of breast milk of mothers vaccinated under different vaccine profiles could be related to the efficacy of each vaccine. We also compared post-vaccination neutralizing antibody results in eight recovered COVID-19 participants. Results for neutralizing antibodies in postvaccinal samples of breast milk showed that 87.5% of the individuals with a previous infection had a positive result of neutralizing antibodies, suggesting that a previous infection to COVID-19 in combination with vaccination can increase the presence of neutralizing antibodies in breast milk.

In this study, different sample collection times after vaccination were used, according to the day reported on which the specific antibodies for each vaccine peaked, or the reports related to the presence of neutralizing antibodies throughout the post-vaccination period. For CanSino, vaccine samples were taken between five and six weeks post-vaccination, with a basis on preceding research on this vaccine [6,26,27], where neutralizing antibodies against SARS-CoV-2 and antibody generation against subunit 1 of protein S (S1 IgG) were reported from week three to week six after vaccination. This suggests that the sample collection time post-vaccination did not play any role in the low SARS-CoV-2 neutralizing antibody response in the CanSino vaccine group obtained in this study.

To our knowledge, this is the first work that reports the neutralizing capacity of anti-SARS-CoV-2 antibodies passed into breast milk from mothers vaccinated against this virus, while most of the studies related to this topic focus on the titers of anti-SARS-CoV-2 antibodies in breast milk of vaccinated mothers. Future studies should also determine and correlate the levels of total IgG, IgA, and sIgA in the breast milk of vaccinated mothers with SARS-CoV-2-neutralizing antibody levels, and evaluate these antibodies against new variants of SARS-CoV-2.

The WHO recommends exclusive breastfeeding for the first six months of life, followed by continued breastfeeding with nutritionally adequate complementary foods for up to two years of age and beyond, with the aim of improving, through optimal feeding, the nutritional status, growth and development, health, and therefore the survival of infants and young children [28]. These recommendations have been supported by a large number of reports on the protective effects of human milk against different infections in infants [11,12,29,30,31]. Our work contributes and supports the knowledge of the presence of protective substances in breast milk, which could be transmitting passive immunity to babies, improving health and survival, especially against a pathogen for which infants cannot yet be directly vaccinated. More studies are needed to evaluate the presence, neutralizing capacity, and duration of the production of antibodies in the breast milk of mothers vaccinated with the different approved vaccines technologies throughout the world.

Some studies have also reported transplacental antibody transfer by the presence of SARS-CoV-2-neutralizing antibody in cord blood of mothers who received the SARS-CoV-2 mRNA vaccines, while no serious complications of pregnancy, delivery, or neonatal outcomes were reported [32,33]. These studies highlight the importance of encouraging vaccination during pregnancy, with evidence that these vaccines can induce antibody responses for pregnant women and their fetuses. The results found in our study underscores the importance to recommend vaccination to pregnant and lactating mothers, and the continuance of breastfeeding to infants due to the potential health benefits (passive immunity against the virus). The importance of these recommendations is highlighted by the absence of an approved vaccine for infants and the continuance emergence of new SARS-CoV-2 strains. To date, passive immunity passed though breast milk is the only known effective line of defense against COVID-19 in infants and should, in our opinion, be recommended by the competent health authorities.

## 5. Conclusions

As vaccination against COVID-19 continues to expand to all populations irrespective of age, sex, race, and other factors, studies on any aspect of lactation or properties of breastmilk from vaccinated or infected mother are greatly lacking. In this research, we show conclusive evidence that the neutralizing capacity of anti-SARS-CoV-2 antibodies passed into breast milk is brand-dependent, with the best results obtained from vaccination with Pfizer and J&J. More importantly, this study shows that the neutralizing capacity of breast milk against COVID-19 could be the only line of defense against the virus in infants for whose age group has not been cleared for vaccination. This emphasizes the importance of recommending the continuance of breastfeeding by the governing authorities to vaccinated lactating mothers.

## Figures and Tables

**Table 1:** Neutralizing Antibodies Titer for all breast milk samples.

Sample	Neutralizing Antibodies Titer				COVID-19 Positive Diagnostic
	Vaccine				
	No Vaccine	Pfizer	J&J	CanSino	
M1		80			
M2				20	
M3				10	
M4				20	
M5		10			
M6		20			+
M7				20	
M8		80			
M9			20		
M10			10		
M11		20			
M12			20		
M13			40		+
M14			40		
M15			40		
M16			40		+
M17			80		
M18		160			
M19		80			+
M20			80		+
M21			20		
M22		80			
M23			40		
M24		40			
M25			20		
M26		80			
M27			40		
M28			40		
M29			40		+
M30			20		+
M31				40	
M32			80		+
M33		40			
M34				20	
M35				20	
M36	20				
M37	20				
M38	20				
Mean	20	63	39	21	
% Samples with NAbs		73%	65%	14%	

If the patient was previously infected with SARS-CoV-2, it is marked with +.

## Data Availability

The data sets generated during the current study are available from the corresponding author on reasonable request.
